# Hierarchical Regression of Wellbeing and Self-Rated Health among Older Adults in Abu Dhabi

**DOI:** 10.3390/ijerph18158006

**Published:** 2021-07-28

**Authors:** Masood A. Badri, Guang Yang, Mugheer Al Khaili, Muna Al Bahar, Asma Al Rashdi, Layla Al Hyas

**Affiliations:** Department of Community Development, UAE University, Abu Dhabi 15551, United Arab Emirates; Guang.Yang@addcd.gov.ae (G.Y.); Mugheer@addcd.gov.ae (M.A.K.); muna.albahar@addcd.gov.ae (M.A.B.); Asma.alrashdi@addcd.gov.ae (A.A.R.); Layla.Alhyas@addcd.gov.ae (L.A.H.)

**Keywords:** self-rated health, older adults, wellbeing, hierarchical regression, Abu Dhabi

## Abstract

This study investigates the wellbeing factors related to self-rated health for older adults in Abu Dhabi (≥55 years). The purpose is to provide a comprehensive analysis of the determinants of self-rated health, considering various wellbeing factors, controlling for factors such as gender, nationality and long-standing illness if present. This research drew from a sample of 2375 older adults who participated in the Abu Dhabi Quality-of-Life Survey (QoL) conducted in 2018. Hierarchical multiple regression analysis was employed where the first two models corresponded to gender, nationality and having a long-standing illness or not. The third model focused on the wellbeing factors of Abu Dhabi citizens (i.e., social networks and connection, income and housing, sports and activities, mental feelings). The analysis revealed the insignificance of gender and nationality as controlled variables while having a long-standing illness showed significant adverse effects. The most significant variables were social support networks, family and social arrangements and connections. Other variables of significance included housing satisfaction, household income satisfaction, frequency of practicing sports, current mental status and life satisfaction. Policymakers could use the outcomes as insider intelligence for policymakers and social work professionals to create policies, programs and services to enhance the lives of older people in Abu Dhabi.

## 1. Introduction

Getting older has become an emergent social concern in Abu Dhabi, United Arab Emirates (UAE) and therefore is relevant for current and future social development policies. The attention to the wellbeing of different categories of residents, especially older adults and the elderlies, has taken center stage attention [1]. Wellbeing has attracted the attention of both policymakers and social service providers. In 2021, the Abu Dhabi government formed the (Quality of Life committee) to “oversee and supervise all affairs of the community in Abu Dhabi in terms of education, health, agriculture, sports, housing, human resources and Emiratization, justice, security and safety, family and childhood, pensions, culture and any other fields defined by the Executive Committee” [2]. The first discussed agenda for the committee focused on health and wellbeing [3]. There is also a growing consensus that the care for older adults goes way beyond merely maintaining their physical health [4]. It is also about maintaining their overall health perception and subjective wellbeing. Therefore, research on self-rated health and QOL for Abu Dhabi’s older adults becomes a central point of strategy and policymaking. The first National Program for Happiness & Wellbeing also stressed the significance of health in wellbeing for all, especially working citizens. Better understanding self-rated health goes along the OECD agenda, which regards the indicator as a holistic reflection and overview of physical and mental health. In addition, self-rated health could provide a holistic overview of both physical and mental health. Despite its subjective nature, the indicator has proved to be a good explainer of future health care needs and mortality [5]. Self-rated health is a personal subjective assessment of the individual status of health. Researchers recognize self-rated health as a reliable variable correlated with many variables of wellbeing for older adults [6,7,8,9,10,11].

The UAE comprises sever Emirates; Abu Dhabi, Dubai, Sharjah, Ras Al Khaimah, Ajman Umm al Qaiwain and Fujairah. However, besides having a unified federal diction-making ministry, each Emirate also has its local government units that set policies and agendas. The QoL initiative is one of the forms of such a specific-Emirate initiative. We should note here that the Abu Dhabi QoL survey uses many health indicators from the OECD Better Life initiatives [12]. Countries differ in defining the age range for the older adults. In Abu Dhabi, the age of 60 is the mandatory retirement age and those who are 60 or above are conventionally considered older adults. In more recent years, the concept of ‘older adults’ (aged 55 or above) has gained popularity in the aging and wellbeing literature [8]. This research adopts the concept of ‘older adults’ (≥55) as it provides adequate and enlarged scope for the old-age issues.

To build a holistic model, we first illustrate the various complex and hypothesized relationships. Then, we use hierarchical regression to identify the effect of a significant cause of self-rated health among older people, which is the presence of long-standing illnesses. For older adults, policymakers should not ignore the fact that many of them have long-standing illnesses. As a result, particular focus should be given to its relevance as it might significantly influence the self-rated health of this segment of community members.

This study aims to identify significant variables affecting the self-rated health of older adults in Abu Dhabi. The presence of a long-standing illness or not and wellbeing as a central focus, this research is set to understand a range of indicators that measure the current self-rated health of the older adults in Abu Dhabi. Thus, it contributes to the body of literature in several ways. First, it provides a comprehensive analysis of the determinants of self-rated health for Abu Dhabi’s older people, considering a variety of wellbeing factors. Second, it offers insights for policymakers and professionals to enhance their understandings of the lives of older adults. Third, policymakers could use the results also as insider intelligence for creating programs to promote and provide convenient services to enhance the lives of the older adults.

## 2. Review of Literature

Self-rated health is a complex measure that inhibits multiple dimensions of the relations between many factors such as physical health and other personal and social features [13,14]. Research has examined self-rated health as a function of other variables or attributes such as age, sex, marital status, educational level, residence, financial source, religious participation, living arrangement, support from family members, community services and medical attributes [6]. Many population surveys worldwide have used self-rated health as an indicator in [15,16,17,18,19] and many studies use a single-item question in relevant surveys to obtain self-rated health [8].

Effective research of the health conditions of older people calls for a detailed understanding of different factors related to various aspects of life, such as demographic and chronic diseases, as well as other wellbeing determinants [20]. Research often addresses self-perceived or self-rated health as a multi-dimensional construct that includes physical, psychological, functional and social variables [19]. Research on self-rated health indicates the significance of some factors such as level of education, sport and recreational physical activity, income, habits, gender, psychological wellbeing and self-esteem [21,22,23,24].

Some studies report a strong association between self-rated health and socioeconomic level [25], where individuals with higher socioeconomic levels tend to report better self-rated health [8]. However, although some research findings indicate that self-rated health assessment decreases with a decreased income level, such an association is not always confirmed [18,26,27]. Other socially related factors include economic and social circumstances over the individual’s life span, making the health of older people even more susceptible to social determination by the accumulation of exposures to risk factors [28].

Social connectedness is a significant factor contributing to various mental and physical health experiences during late adulthood. Relevant studies note that amongst other factors, lack of social ties is associated with increased depression and poorer overall health [29,30]. On the other hand, positive social ties provide health advantages, including fewer physical and mental health problems [30,31]. Some investigated the association between social connectedness, emotional wellbeing and self-rated health among older adults and confirmed a positive association between self-rated health and social connectedness items such as social network characteristics, family and friend support and social ties with neighbors [32]. Other family attributes and aspects of social connectedness also play significant roles in explaining self-rated health. Many studies reported the importance of living arrangements and the number of people living with older adults [33,34].

The role of sport and recreational physical activity has received much attention. Some noted that men indicating no leisure-time physical activity more frequently described their health as poor or very poor than men with a satisfactory level of recreational physical activity [35]. The same pattern was observed for women, but it was not statistically significant. Meanwhile, a study by the University of Lodz noted that a sufficient level of leisure-time physical activity beneficially affects self-perception of health [27]. Research results, in general, have been consistent in indicating a strong association between lack of sport/physical activity and lower self-rated health [36].

Some researchers examined the relationship between self-rated health and life satisfaction while testing the moderating effect of physical activity [37,38]. Results showed positive relationships and significant interaction effects between physical activity and self-rated health and life satisfaction.

Some studies focused on the effect of religious involvement on self-rated health. In general, results showed that spiritual and religious involvement is associated with physical and mental health [39,40,41,42,43,44]. Subjective mental variables (i.e., feeling psychologically worse) directly and indirectly affect self-rated health [43]. Some note that depression may involve cognitive processing, causing individuals to portray and manifest lower satisfaction with their lives and, consequently, lower self-rated health [23,45].

Some studies examine gender differences in the association between work-family conflict and self-rated health and evaluate the effect of educational attainment. A study noted that women’s educational level interacted with work-family conflict indicators [46]. For time-based effects of work on the family, highly educated women had higher odds of suboptimal self-rated health. Regarding gender, some studies note that women report more negative self-rating of health due to the proportion of health problems [46].

Prior awareness of an illness influences the judgment the individual has of self-rated health [47]. Some add that such knowledge of potential long-standing might lead to biases regarding self-rated health [48,49,50]. Such biases might affect self-rated health significantly [8,24].

According to the World Health Organization (WHO) [41], housing can be considered on a four-level scale, i.e., the physical structure, the psychosocial, economic and cultural construction created by the household and the immediate housing environment, and the community environment. Concerning housing scales and self-rated health, some found housing insignificant. In contrast, other studies found some relations when they analyzed the associations among housing type, neighborhood safety behavior, self-rated health and psychological wellbeing [51].

By including gender, nationality, prior long-standing illnesses and a range of wellbeing determinants, this study tested the relationships between these variables and subjective self-rated health, drawing on a sample of older people in Abu Dhabi.

## 3. Methods

### 3.1. Study Sample of Older People and Measurements

The QoL survey covered various dimensions and factors that affect the wellbeing of residents in Abu Dhabi. The survey utilized inputs from many international social surveys and studies. These surveys included the OECD’s Better Life [12], Gallup Global Wellbeing Survey [52], World Happiness Report [53] and European Quality of Life Survey [54]. Those dimensions included housing, household income and wealth, jobs and earnings, work-life balance, health, education and skills, personal safety and security, social and cultural values, social connections, civic engagement and governance, environmental quality, social and community services, access to information (internet and Wi-Fi) and subjective wellbeing measures of happiness and life satisfaction. 

A pre-analysis meeting of the researchers and selected community policymakers produced a list of few biographic variables that should be considered for the self-rated health of older adults in Abu Dhabi. The primary variable identified was (gender, nationality and having or not having longstanding illness). Further extensive statistical analysis that included correlation analysis, simple regression analysis, multicollinearity analysis identified a final list of 13 variables. The scales used in the survey were different for each of the variables are provided here:

- Having a longstanding illness or not (Binary yes and no).
- Overall housing satisfaction (1–5 scale).
- Satisfaction with household income (1–5 scale).
- Frequency of practicing sport (1–5 scale).
- Frequency of having lots of energy (1–5 scale).
- Frequency of feeling downhearted and depressed (1–5 scale).
- Size of support network (1–5 scale).
- Satisfaction with family life (1–5 scale).
- Life satisfaction (0–10 scale).
- Frequency of having informal activities with friends (1–6 scale).
- Relationships with other acquainted people (1–5 scale).
- The number of family members living together (continuous scale starting from zero).
- Importance of maintaining family ties (1–5 scale).

The systematic random sampling survey covered all regions of the emirate of Abu Dhabi and resident categories aged 15 and above in Abu Dhabi. The survey was administered online and sent to residents of Abu Dhabi. Respondents came from large databases of public and private entities. For continuous follow-up of the response, the feedbacks and examined the separate internet links for each entity (or department) database. For workers residing in residential worker cities, a team of research assistants from the Statistics Center Abu Dhabi entered residential worker cities to collect responses using laptops. To provide adequate representations of the various respondent categories, we used sampling weights. 

### 3.2. Analysis Methods

The primary purpose of the research is to provide a comprehensive view of the determinants of self-rated health for Abu Dhabi’s older adults (≥55 years). The objectives aim to consider a variety of wellbeing factors, controlling for factors such as gender, nationality and long-standing illness if present. Such objectives entail the consideration of various wellbeing factors. The main aim is to understand the complex nature of those combinations that consider a person’s physical, mental, emotional, economic, social and health factors. Hierarchical multiple regression analysis was employed where the levels corresponded to gender, nationality and having a long-standing illness or not. The ultimate objective is to provide some outcomes that could be used as insider intelligence for decision-makers to enhance the lives of older people in Abu Dhabi.

The analysis uses (SPSS, version 22, IBM Corp, Armonk, New York, USA). We first examined how age influenced self-rated health. Next, we calculated the means of self-rated health (on a scale of 1–5) and age categories to see the overall scores of self-rated health. Descriptive measures of self-rated health by gender, nationality and having a long-standing illness or not were also analyzed. Then, after standardizing the data, hierarchical multiple linear regressions were run with variables of gender, nationality and having a long-standing illness or not. Next, we added a list of variables from different wellbeing domains in the third model, including housing, income and earnings, jobs, health, education, safety and security, environment, social connection, community and civic involvement and social and cultural values. Finally, the analysis used significance tests for change in R-square to assess the degree to which additional variables accounted for the variance in self-rated health.

### 3.3. Regression Assumptions

Regression analysis requires some assumptions to be true. We could check these assumptions as part of the multiple regression procedure, except for checking the normal distribution of the dependent variable. The Shapiro–Wilk significance test produced a value of 0.3948, which is not statistically significant. Such a result is a requirement for normality. The Kolmogorov–Smirnov significance is 0.0983, which is again an indication of non-significance. Since both were not significant, we concluded that the dependent variable is normally distributed.

Concerning sample size, the number of 2375 respondents in the analysis was deemed sufficient given the explanatory variables in the model. Furthermore, normality tests verified that the dependent variable is normally distributed. Regarding the residual statistics, the standard residuals fall within a minimum of (−2.861) and a maximum of (2.749). Both are within the range of −3 and +3.

Cook’s distance is used in regression analysis to find influential outliers in a set of variables. The values of Cook’s distance range from the minimum of 0.000 to the maximum of 0.327. Cook’s values identified few outliers that we removed accordingly. These steps produced a sample of older adults to be 2347. In addition, we recorded the highest correlation to be 0.510. As a result, all correlations were within the allowable limits concerning multicollinearity. Thus, results indicate the absence of multicollinearity between the variables.

## 4. Results

A total of 45,005 residents participated in the survey. More than 3000 fell into the age category of 55 and above. However, only 2375 provided complete responses to all variables in the model. Among this group of older adults, 54.7% were males; those with a long-standing illness accounted for 35.5%; the majority (86.7%) were married; 73.5% were UAE nationals. The Abu Dhabi Emirate is divided into three regions: Abu Dhabi, Al Ain and Al Dhafra. About 74.6% of the respondents resided in the Abu Dhabi region, 18.9% in the Al Ain region and 6.6% in the Al Dhafra region. Concerning educational attainment, 38.7% held equivalent college degrees, 27.6% held post-graduate degrees, 12.4% had college diplomas, 10.2% had below secondary school degrees and 7.1% held secondary school degrees. For older people 55 years or older, on a scale of (1–5), they recorded a mean score of (3.185). Further statistical tests showed no evidence to suggest that the samples in different categories had different distributions. Table 1 provides a summary of the description of sample participants.

Table 2 provides a general overview of the self-rated health by different categories of older adults. On average, most of the respondents reported a ‘good’ or better health status. The percentages of females recording a ‘poor’ and ‘fair’ health status are higher than their male counterparts. The proportions of UAE nationals who fall in the ‘poor’ and ‘fair’ categories are lower than expatriates. Those with a long-standing illness recorded the highest percentage (6.69%) in the ‘poor’ category while recording the lowest in the ‘very good and ‘excellent’ categories of self-rated health.

We selected self-rated health to be the dependent variable. For the first model, we entered gender and nationality (UAE national or non-UAE national). For the second model, we entered the dummy variable of having a long-term health problem or not (‘yes’ coded as one and ‘no’ as zero). Finally, for the third model, the primary wellbeing variables of interest were entered to understand their influence capacity above the variables in the first two models.

Table 3 presents the model summary of the R-square, Adjusted R-square and R-square changes associated with each step in the hierarchical regression. The first model with an R-square of 0.017 suggests that gender and nationality only account for 1.7 percent of the variance in self-rated health. The Adjusted R-square is also too small to be considered. The F-change (from 0 to 1.263) for model 1 is not significant. The ANOVA table (Table 4) confirms the insignificance of model 1. 

The results of model 2 indicate a noticeable improvement, where R increases from 0.130 in model 1 to 0.453. The R-square value of 0.205 suggests that model 2 accounts for 20.5% of the variability of the dependent variable; the F value changes (from 1.263 to 17.292). The associated regression coefficient for ‘having a long-standing illness or not’ is (−0.767).

In Model 3, we entered the variables associated with wellbeing. The R-value increased from 0.453 to 0.918 and the R-square from 0.205 to 0.842. Such results suggest that model 3 can account for 84.2% of the variance in self-rated health by older adults. However, not all the variables in model 3 are significant. Table 4 shows the specifics regarding each regression model and the associated residuals. For better illustration, Table 5 shows the coefficients of the significant variables included in the models. A total of 12 variables in model 3 are significant. Variables related to social connection and support (family or friends) provide six variables. Those variables are the size of support, satisfaction with family life, the frequency of having informal activities with friends, number of family members living together, relationships with other acquainted people and importance of maintaining family ties. Results also show that two variables related to mental feelings are significant (frequency of energy and feeling downhearted/depressed). Two variables related to more material needs are significant (satisfaction with household income and overall housing satisfaction). Being more active regarding sports provide a significant t-value too. The final variable is related to overall life satisfaction.

## 5. Discussions

The relationships between older people’s self-rated health and other wellbeing variables are the focus of this research. However, other variables might act with some influence above and beyond the individual factors of wellbeing. Hierarchical regression analysis with its unique sequence can identify the unique effect of each wellbeing factor included in the model. The analysis used a nested modeling procedure where the first model is nested in model two and model two is nested in model three.

The research assessed the influence of many indicators on self-rated health among older people in Abu Dhabi, using a single item of self-rated health. The research examined the relations between self-rated health and other personal and family-specific correlates of wellbeing among older people in Abu Dhabi. The study recognized that self-rated health and related factors of emotional feelings might be distinct subjective wellbeing components [55]. It might be shaped differently depending on demographic factors such as age [56,57]. Similar studies elsewhere found that self-reported health provides a good measure compared to other objective and subjective measures of wellbeing such as happiness and satisfaction with income [58,59]. Based on the assumptions derived from other research that both personal factors influence self-rated scores of health in people’s lives such as marriage and work and community and societal circumstances as well [60], this present research added in the analysis wellbeing factors such as feeling depressed, income, housing satisfaction, physical activity, economic factors, living arrangements and social connections.

The analysis firstly revealed the insignificance of gender and nationality as controlled variables. Furthermore, consistent with other research [25], it reflected the significance of a mixture of socioeconomic factors. Overall, the research supported self-rated health and some social, economic, cultural and life factors. The results of this study supported other research [28] by stressing that the self-rated health of older people is even more susceptible to other social determinants. It also stressed that self-rated health is a multidimensional construct that includes physical, psychological, functional and social variables [19]. The study confirmed the significance of sport and recreational physical activity, income, psychological wellbeing and self-esteem and family and friendships [21,23,24].

Pre-analysis of the Abu Dhabi Quality of Life Survey data showed that age could significantly explain self-rated health. Those aged 55 and older reported the lowest scores. However, some studies in China and Japan mostly found no significant effect on self-rated health [6]. Thus, the Abu Dhabi finding may suggest that older adults might experience different wellbeing circumstances (i.e., social connection and support, mental health, life satisfaction, sports and activities, housing and income satisfaction).

Concerning gender, some studies note that older women report more negative self-rating of health due to the proportion of health problems [47]. The results of this present Abu Dhabi study conveyed that older women were not likely to be different from older men when it comes to self-rated health. The findings also contradicted other studies conducted in other communities and cultures worldwide where gender was a significant factor [23,25,61,62]. However, those studies also add that we should not ignore the mediating role of some factors when explaining self-rated health. In Abu Dhabi, both older women and men receive the same health services and attention no matter what region they live in. There is also no reason to doubt that in Abu Dhabi, both older women and men enjoy the same living circumstances regarding social connections and family relations.

The study shows the association between self-rated health and social connectedness (i.e., family and friend relations and support and social ties) and emotional wellbeing. Social connectedness items were positively associated with self-rated health. Social connectedness shared the highest number of variables that positively affected self-rated health among older adults in Abu Dhabi. Results are consistent with the findings reported by others [30]. The emotional wellbeing themes reflected by the Abu Dhabi study included mental feelings of having lots of energy and feeling downhearted and depressed. Consistent with other empirical findings [29,31,32,34], this research showed that poor quality social ties are associated with increased levels of depression and poorer overall self-rated health. The results are consistent with some research [23,45,49] to point out that the related mental feelings variables (i.e., frequency of having lots of energy and frequency of feeling downhearted/depressed) are related to self-rated health among older adults in Abu Dhabi. Feeling downhearted and depressed produced a significant adverse effect on the self-rated health of older adults in Abu Dhabi. 

This research produced evidence to suggest that prior awareness of an illness, such as a long-standing disease, might be related to the individual’s judgment of self-rated health. Consistent with other research [21], knowledge of a potential long-standing disease may have more significant relations with the individual and lead to biases regarding self-rated health. The presence of such risk factors for chronic diseases decreases self-rated health significantly. On the other hand, the sport and physical activity-related factors function as essential variables in reflecting self-rated health amongst older adults, which is in line with the results of some international studies [35,36,38]. 

The results also illustrated the significant role of satisfaction with household income and satisfaction with housing-related to self-rated health. Thus, results are consistent with the results of other studies [63,64]. However, the result is inconsistent with other international replications that did not confirm such association concerning older adults [18,26,27,56,64,65]. 

Finally, in line with other research [37], this Abu Dhabi study found significant relation of overall life satisfaction on self-rated health among older people. However, contrary to what most believe and other research about religious involvement and self-rated health [43], the effect of religious involvement on self-rated health was not supported by this research. 

## 6. Conclusions

This research validates and confirms the multidimensional nature of self-rated health amongst older people and argues that self-rated health is complex and might inhibit multiple relationships and interactions between many factors. The study reveals several significant factors related to older adults’ self-rated health in Abu Dhabi. For example, a significant factor is related to having a long-standing illness or not. Other wellbeing factors include frequency of practicing sports, frequency of having informal activities with friends, relationships with other people, the number of family members living together, family and social arrangements and connections-related variables, overall satisfaction with current residence, satisfaction with household income, feeling downhearted and depressed and life satisfaction. 

Policymakers could use the results of this study as insider intelligence for creating programs to promote and provide convenient services to enhance the lives of older people. For policymakers, results point to the significant role of family and friends’ ties in self-rated health. For policy implications, it is necessary to understand better how relationship status and social ties might be associated with self-rated health and wellbeing among older adults. The Quality of Life committee in Abu Dhabi could use results to better propose and guide policies that promote wellbeing of older adults. 

Future research should focus more on investigating the association between social connectedness, emotional wellbeing and self-rated health among elderlies and what we could consider enriching using other experiences or interferences. Future research could also consider including employment and retirement-related variables as extra levels in the hierarchical analysis. A few international studies found significant relationships between continued employment after retirement and self-rated health [5]. Work and professional duties might help older adults maintain their self-esteem, connectedness and sense of belonging, which may profoundly affect health. As research limitations and challenges, to a certain extent, this research indicates that relevant socio-economic factors drive possible biases in self-rated health. We should emphasize that the analytical approach employed by this study partly addresses the problem as the estimated hierarchical regression models include a rich set of controls for such factors. However, despite specifying a detailed model of self-rated health, the analysis does not imply a causal effect of those wellbeing variables on self-rated health status. Future research should focus on increasing the sample size of older adults. In addition, future research should focus on a more proper representation of older adults in Abu Dhabi (i.e., gender, family size, nationalities, employment status, type of housing).

## Figures and Tables

**Table 1:** Description of sample participants.

Characteristics	Sample Percent
**Gender**	
Male	54.7%
Female	45.3%
**Marital status**	
Single	9.4%
Married	86.7%
Divorced	3.9%
**Education**	
Below secondary	10.2%
Secondary	7.1%
College diploma	12.4%
College bachelor’s degree	38.7%
Masters/Doctoral degree	27.6%
**Location**	
Abu Dhabi	74.6%
Al Ain	18.9%
Al Dhafra	6.6%
**Nationality**	
Emirati	73.5%

**Table 2:** General descriptive outcomes of self-rated health.

Categories	Poor	Fair	Good	Very Good	Excellent
Total	3.03%	17.57%	43.27%	26.52%	9.61%
Male	2.64%	16.24%	44.90%	26.65%	9.58%
Female	4.16%	21.39%	38.61%	26.14%	9.70%
UAE national	2.78%	16.75%	43.15%	27.03%	10.28%
Non-UAE national	3.75%	19.92%	43.59%	25.05%	7.69%
Have long-standing illness	6.69%	34.59%	43.17%	12.94%	2.62%
Does not have long-standing illness	0.61%	7.88%	42.68%	35.67%	13.16%

**Table 3:** Hierarchical multiple regression model summary.

Model	R	R Square	Adjusted R Square	Std. Error of the Estimate	R Square Change	F Change	df1	df2	Sig. F Change
1	0.130	0.017	0.003	0.953	0.017	1.263	1	74	0.265
2	0.453	0.205	0.183	0.863	0.188	17.29	1	73	0.0001
3	0.918	0.842	0.748	0.479	0.637	7.306	26	47	0.0001

**Table 4:** ANOVA results.

Model	Sum of Squares	d.f.	Mean Square	F	Sig.	Sum of Squares
1	Regression	1.148	1	1.148	1.263	0.265
	Residual	67.273	74	0.909		
	Total	68.421	75			
2	Regression	14.032	2	7.016	9.417	0.000
	Residual	54.389	73	0.745		
	Total	68.421	75			
3	Regression	57.633	28	2.058	8.967	0.000
	Residual	10.789	47	0.230		
	Total	68.421	75			

**Table 5:** Model coefficients.

	Unstandardized Coefficients		Standardized Coefficients		
	B	Std. Error	Beta	t	Sig.
**Model 1**					
(Constant)	3.727	0.382	---------	9.755	0.001
Gender	−0.364	0.324	−0.130	−1.124	0.265
Nationality	0.029	0.064	0.016	0.462	0.644
**Model 2**					
(Constant)	4.041	0.354	---------	11.414	0.001
Gender	−0.313	0.293	−0.112	−1.069	0.289
Nationality	0.144	0.058	0.076	1.426	0.115
Having a long-standing illness or not	−0.767	0.056	−0.431	−13.806	0.000
**Model 3**					
(Constant)	−1.297	0.174	---------	7.470	0.000
Gender	0.003	0.072	0.001	0.047	0.963
Nationality	0.118	0.052	0.063	1.256	0.124
Having longstanding illness or not	−0.578	0.050	−0.325	−11.551	0.001
Overall housing satisfaction	0.058	0.025	0.066	2.274	0.023
Satisfaction with household income	0.108	0.022	0.150	2.997	0.005
Frequency of practicing sport	0.079	0.016	0.136	4.825	0.001
Frequency of having lots of energy	0.237	0.030	0.234	7.829	0.001
Frequency of feeling downhearted/depressed	−0.124	0.131	−0.127	2.331	0.024
Size of support network	0.057	0.020	0.083	2.891	0.004
Satisfaction with family life	0.411	0.178	0.229	2.682	0.009
Life satisfaction	0.041	0.015	0.085	2.762	0.006
Frequency having informal activities (friends)	−0.286	0.071	−0.368	−4.028	0.001
Relationships with other acquainted people	0.600	0.158	0.297	3.798	0.001
Number of family members living together	0.061	0.016	0.278	3.725	0.001
Importance of maintaining family ties	0.480	0.189	0.241	2.539	0.014

## Data Availability

The data presented in this study are available on request from the corresponding author. The data are not publicly available due to privacy restrictions.

## References

[1] Godchaux-Berezhnova I., Moonesar I. (2021). Using Global Practices & Policies to Inform the UAE Quality of Life & Wellbeing in the 21st Century. Mohammed Bin Rashid School of Government POLICY BRIEF, Policy Brief No. 56. https://mbrsgcdn.azureedge.net/cmsstorage/mbrsg/files/7b/7b907250-3c84-4dee-b2d5-9176e2e6f8a4.pdf.

[2] Abu Dhabi Executive Council (2021). Decree Forming the Quality of Life and Wellbeing for Abu Dhabi. https://wam.ae/en/details/1395302835611.

[3] Department of Community Development (2021). Meeting Agenda—Good Health and Wellbeing. https://u.ae/en/about-the-uae/leaving-no-one-behind/3goodhealthandwellbeing.

[4] Badri M., Al Khaili M., Al Bahr M., Guang Y., Al Rashdi A. Elderly happiness—A path analytic model exploration of determinants of wellbeing in Abu Dhabi. Proceedings of the Asian Conference on Aging & Gerontology (AGen2019).

[5] Palladino R., Tayu Lee J., Ashworth M., Triassi M., Millett C. (2016). Associations between multimorbidity, healthcare utilisation and health status: Evidence from 16 European countries. Age Ageing.

[6] Wang N., Motoki I., Tetsuya O., Rumiko H., Hiroko M., Liu X., Sasazawa Y., Shosuke H., Tetsuo S. (2005). Perceived health as related to income, socio-economic status, lifestyle, and social support factors in middle-aged Japanese. J. Epidemiol..

[7] Sun W., Watanabe M., Tanimoto Y., Shibutani T., Kono R., Saito M., Usuda K., Kono K. (2007). Factors associated with good self-rated health of non-disabled elderly living alone in Japan: A cross-sectional study. BMC Public Health.

[8] Damian J., Pastor-Barriuso R., Valaderama-Gama E. (2008). Factors associated with self-rated health in older people living in institutions. BioMed Cent..

[9] Darviri C., Fouka G., Gnardellis C., Artemiadis A.K., Tigani X., Alexopoulos E.C. (2012). Determinants of self-rated health in a representative sample of a rural population: A cross-sectional study in Greece. Int. J. Environ. Res. Public Health.

[10] Beenackers A., Kamphuis C.B., Giskes K., Brug J. (2012). Socioeconomic inequalities in occupational, leisure-time, and transport-related physical activity among European adults: A systematic review. Int. J. Behav. Nutr. Phys. Act..

[11] Santiago L.M., Novaes C.D.O., Mattos I.E. (2010). Self-rated health (SRH) as a predictor of mortality in elderly men living in a medium-size city in Brazil. Arch. Gerontol. Geriatr..

[12] Organization for Economic Co-operation and Development (OECD) (2020). Measuring Wellbeing and Progress. https://www.oecd.org/sdd/OECD-Better-Life-Initiative.pdf.

[13] Roos N., Havens B. (1991). Predictors of successful aging: A twelve-year study of Manitoba elderly. Am. J. Public Health.

[14] Mor V., Wilcox V., Rakowski W., Hiris J. (1994). Functional transitions among the elderly: Patterns, predictors, and related hospital use. Am. J. Public Health.

[15] Paradies Y. (2006). A systematic review of empirical research on self-reported racism and health. Int. J. Epidemiol..

[16] Fan A., Strasser S., Zhang X., Fang J., Crawford C. (2015). State socioeconomic indicators and self-reported hypertension among US adults—2011 Behavioral Risk Factor Surveillance System. Prev. Chronic Dis..

[17] Kunst A., Bos V., Lahelma E., Bartley M., Lissau I., Regidor E. (2005). Trends in socioeconomic inequalities in self-assessed health in 10 European countries. Int. J. Epidemiol..

[18] Kunst A., Geurts J., van den Berg J. (1995). International variation in socioeconomic inequalities in self-reported health. J. Epidemiol. Community Health.

[19] Millán-Calenti J.C., Sánchez A., Lorenzo T., Maseda A. (2012). Depressive symptoms and other factors associated with poor self-rated health in the elderly: Gender differences. Geriatr. Gerontol. Int..

[20] Campos A., Albala C., Lera L., Sánchez H., Vargas A., Ferreira E. (2015). Gender differences in predictors of self-rated health among older adults in Brazil and Chile. BMC Public Health.

[21] Carlson P. (2001). Risk behaviors and self-rated health in Russia. J. Epidemiol. Community Health.

[22] Kaleta D., Polańska K., Dziankowska-Zaborszczyk E., Hanke W., Drygas W. (2009). Factors influencing self-perception of health status. Central Eur. J. Public Health.

[23] Ocampo J. (2010). Self-rated health: Importance of use in elderly adults. Colomb. Médica.

[24] Piko B. (2000). Health-related predictors of self-perceived health in a student population: The importance of physical activity. J. Community Health.

[25] Fernández B., Rosero-Bixby L., Koivumaa-Honkanen H. (2016). Effects of self-rated health and self-rated economic situation on depressed mood via life satisfaction among older adults in Costa Rica. J. Aging Health.

[26] Erginoz E., Alikasifoglu M., Ercan O., Uysal O., Ercan G., Albayrak-Kaymak D. (2004). Perceived health status in a Turkish adolescent sample: Risk and protective factors. Eur. J. Pediatrics.

[27] Kaleta D., Makowiec-Dąbrowska T., Jegier A. (2004). Leisure-time physical activity, cardiorespiratory fitness and work ability: A study in randomly selected residents of Lodz. Int. J. Occup. Med. Environ. Health.

[28] Geib L.T.C. (2012). Social determinants of health in the elderly. Cien. Saude Colet..

[29] Brummett B.H., Barefoot J.C., Siegler I.C., Clapp-Channing N.E., Lytle B.L., Bosworth H.B., Williams R.B., Mark D.B. (2001). Characteristics of socially isolated patients with coronary artery disease who are at elevated risk for mortality. Psychosom. Med..

[30] Cornwell B., Schumm L.P., Laumann E.O., Kim J., Kim Y. (2014). Assessment of social network change in a longitudinal survey. J. Gerontol. Ser. B.

[31] Fiori K.L., Antonucci T.C., Cortina K.S. (2006). Social network typologies and mental health among older adults. J. Gerontol. Ser. B.

[32] Armer A., Proulx C. (2019). Associations between social connectedness, emotional wellbeing, and self-rated health among older adults: Difference by relationship status. Res. Aging.

[33] Bongaarts J., Zimmer Z. (2002). Living arrangements of older adults in the developing world: An analysis of demographic and health survey household surveys. J. Gerontol. Ser. B.

[34] Xu D., Arling G., Wang K. (2019). A cross-sectional study of self-rated health among older adults: A comparison of China and the United States. BMJ Open.

[35] Nowak M. (2006). Factors determining physical fitness self-evaluation and health self-evaluation in physically active women. New Med..

[36] Kwaśniewska M., Bielecki W., Drygas W. (2004). Sociodemographic and clinical determinants of quality of life in urban population of Poland. Cent. Eur. J. Public Health.

[37] Marques A., Peralta M., Gouveia E., Chávez F., Valeiro M. (2018). Physical activity buffers the negative relationship between multi-morbidity, self-rated health and life satisfaction. J. Public Health.

[38] Maher J., Pincus A., Ram N. (2015). Daily physical activity and life satisfaction across adulthood. Dev. Psychol..

[39] Chirico F., Sharma M., Zaffina S., Magnavita N. (2020). Spirituality and prayer on teacher stress and burnout in an Italian cohort: A pilot, before-after controlled study. Front. Psychol..

[40] Chirico F., Nucera G. (2020). An Italian experience of spirituality from the Coronavirus Pandemic. J. Relig. Health.

[41] Chirico F. (2016). Spiritual wellbeing in the 21st century: It is time to review the current WHO’s health definition. J. Health Soc. Sci..

[42] Ardelt M. (2003). Effects of religion and purpose in life on elders’ subjective wellbeing and attitudes toward death. J. Relig. Gerontol..

[43] Chatters L.M., Bullard K.M., Taylor R.J., Woodward A.T., Neighbors H.W., Jackson J.S. (2008). Religious participation and DSM-IV disorders among older African Americans: Findings from the National Survey of American Life (NSAL). Am. J. Geriatr. Psychiatry.

[44] Krause N., Hayward R.D. (2014). Religious involvement, practical wisdom, and self-rated health. J. Aging Health.

[45] Anstey K., Luszcz M., Giles L., Andrews G. (2001). Demographic, health, cognitive, and sensory variables as predictors of mortality in very old adults. Psychol. Aging.

[46] Griep R., Toivanen S., van Diepen C., Guimarães J., Camelo L., Juvanhol L., Aquino E., Chor D. (2016). Work-family conflict and self-rated health: The role of gender and educational level. Baseline data from the Brazilian Longitudinal Study of Adult Health (ELSA-Brasil). Int. J. Behav. Med..

[47] Jürges H. (2007). True health vs response styles: Exploring cross-country differences in self-reported health. Health Econ..

[48] van der Made C.I., Simons A., Schuurs-Hoeijmakers J., van den Heuvel G., Mantere T., Kersten S., van Deuren R.C., Steehouwer M., van Reijmersdal S.V., Jaeger M. (2020). Presence of genetic variants among young men with severe COVID-19. JAMA.

[49] Lavretsky H., Kumar A. (2002). Clinically significant non-major depression: Old concepts, new insights. Am. J. Geriatr. Psychiatry.

[50] Singh-Manoux A., Martikainen P., Ferrie J., Zins M., Marmot M., Goldberg M. (2006). What does self-rated health measure? Results from the British Whitehall II and French Gazel cohort studies. J. Epidemiol. Community Health.

[51] Berglund E., Westerling R., Lytsy P. (2017). Housing type and neighborhood safety behavior predicts self-rated health, psychological wellbeing and frequency of recent unhealthy days: A comparative cross-sectional study of the general population in Sweden. Plan. Pract. Res..

[52] Gallup Global (2020). Gallup Global Wellbeing. https://news.gallup.com/poll/126965/gallup-global-wellbeing.aspx.

[53] World Happiness (2020). World Happiness Report. https://happiness-report.s3.amazonaws.com/2020/WHR20.pdf.

[54] Eurofound (2020). European Quality of Life Survey (EQLS). https://www.src.isr.umich.edu/international/european-quality-of-life-survey-eqls/.

[55] Graham C.L., Nikolova M. (2015). Bentham or Aristotle in the development process? An empirical investigation of capabilities and subjective wellbeing. World Dev..

[56] Ford J., Spallek M., Dobsin A. (2008). Self-rated health and a healthy lifestyle are the most important predictors of survival in elderly women. Age Ageing.

[57] Flores G., Ingenhaag M., Maurer J. (2015). An anatomy of old-age disability: Time use, affect and experienced utility. J. Health Econ..

[58] Jagodzinski W. (2010). Economic, social, and cultural determinants of life satisfaction: Are there differences between Asia and Europe?. Soc. Indic. Res..

[59] Lou V.W.Q. (2010). Life satisfaction of older adults in Hong Kong: The role of social support from grandchildren. Soc. Indic. Res..

[60] Diener E., Inglehart R., Tay L. (2013). Theory and validity of life satisfaction scales. Soc. Indic. Res..

[61] Alarcão V., Guiomar S., Oliveira A., Severo M., Correia D., Torres D., Lopes C. (2020). Food insecurity and social determinants of health among immigrants and natives in Portugal. Food Secur..

[62] Zhou X., Hetrick S.E., Cuijpers P., Qin B., Barth J., Whittington C.J., Cohen D., Del Giovane C., Liu Y., Michael K.D. (2015). Comparative efficacy and acceptability of psychotherapies for depression in children and adolescents: A systematic review and network meta-analysis. World Psychiatry.

[63] Bonner W., Weiler R., Orisatoki R., Lu X., Andkhoie M., Ramsay D., Yaghoubi M., Steeves M., Szafron M., Farag M. (2017). Determinants of self-perceived health for Canadians aged 40 and older and policy implications. Int. J. Equity Health.

[64] Nayak S., Hubbard A., Sidney S., Syme S.L. (2016). Characteristics associated with self-rated health in the CARDIA study: Contextualizing health determinants by income group. Prev. Med. Rep..

[65] Maniscalco L., Miceli S., Bono F., Matranga D. (2020). Self-perceived health, objective health, and quality of life among people aged 50 and over: Interrelationship among health indicators in Italy, Spain, and Greece. Int. J. Environ. Res. Public Health.

